# Knowledge, Attitude, and Practice of Yoga in Rural and Urban India, KAPY 2017: A Nationwide Cluster Sample Survey

**DOI:** 10.3390/medicines7020008

**Published:** 2020-02-05

**Authors:** Amit S Mishra, Rajesh SK, Vadiraja HS, Raghuram Nagarathna, Akshay Anand, Himshikha Bhutani, Madhava Sai Sivapuram, Amit Singh, Hongasandra Ramarao Nagendra

**Affiliations:** 1 Swami Vivekananda Yoga Anusandhana Samsthana, Bengaluru 560019, India; dirghayuh@gmail.com (A.S.M.); dramits90@gmail.com (A.S.);; 2 Neuroscience Research Lab, Department of Neurology, Postgraduate Institute of Medical Education and Research, Chandigarh 160012, India; himshikha.bhutani@gmail.com; 3 Department of General Medicine, Dr. Pinnamaneni Siddhartha Institute of Medical Sciences and Research Foundation, Chinna-Avutapalli 521101, India; madhavasai2011@gmail.com

**Keywords:** yoga, health, lifestyle, integrative medicine

## Abstract

**Background:** To examine the knowledge, attitudes, and practice gap of yoga across India based on implicit perceptions. **Methods:** The present study is a nationwide door-to-door survey conducted using a questionnaire/screening form. The data were collected from a national survey conducted under the *Niyantrit Madhumeh Bharat* (NMB) program initiated by The Ministry of Ayurveda, Yoga, Unani, Siddha, Homeopathy (AYUSH), Government of India, from all major zones of the country. A total of 162,330 participants who joined the NMB program were recruited in our study. Results: Out of the total respondents to the survey, it was observed that 11.8% [13,336/112,735] practice yoga, which was highest in the north zone [4,567/112,735] and lowest in the east zone [971/112,735]. Out of 101,643 respondents, 94,135 of the individuals who participated in the survey believed that yoga improved their lifestyle, and 90,102/98,518 believed that yoga prevented diabetes, revealing a huge knowledge–practice gap. **Conclusions:** The scale of the knowledge–practice gap coupled with the general acceptability of yoga calls for a change in the conventional healthcare provisions by its integration with modern medicine. The population-wide positive perceptions about yoga as a preventive health tool can not only catalyze consensus disease-specific yoga modules but also bridge the knowledge–practice gap that exists because of limited yoga centers and professionals.

## 1. Introduction

The latest ‘global action plan on physical activity’, by the WHO (World Health Organization) stresses health and wellness as an outcome of being ‘active’ [1]. Health is a broad concept, finding its roots in physical, mental, social, and spiritual wellness [2]. In this context, public health delivery is critical for provisioning an evidence-based integration of alternative approaches for the public good. This is possible by analyzing health-seeking behavior based on the knowledge–practice gap in the population (a knowledge–practice gap is defined as the failure of the public to adopt the existing practices despite knowledge that it promotes the health of an individual). As an integrative medicine (IM) approach that incorporates a broad range of therapies for holistic health based on preferences for Western and traditional medicine and has shown varying acceptability, there is a need for national survey for understanding the knowledge–practice gap in popular health promotion activities such as yoga in a population where yoga originated. To exemplify, a study conducted in Hong Kong by Wong et al. revealed that 40% of people turned to Traditional Chinese Medicine (TCM) mostly as a second option [3]. Similarly, a Taiwan based study by Ma et al. depicted a very low prevalence of TCM among children with asthma, with almost all parents opting for Western medicine [4]. Among other mind–body techniques, T’ai chi/Qigong practice was reported to have a lifetime prevalence of 3.1% in the United States of America (U.S.A) [5]. Similarly, the data collected in Australia over 10 years (2001–2010) showed a 3% prevalence rate for yoga/pilates, 0.6% for T’ai chi/Qigong, and 19.2% for other fitness activities across the country [6]. In contrast, 84% of the physicians in Japan use Kampo, a traditional Japanese medicine, in their practice and practice acupuncture (a TCM), which is accepted across 103 countries with 29 countries having regulators for acupuncture in the world [7]. This calls for the need to integrate the traditional medicine/complementary medicine with modern medicine in the world. The importance of existing traditional/complementary medicine can be highlighted by integrating it with modern medicine as required for universal health coverage (UHC). The WHO is also working toward bringing traditional/complementary medicine into national policies for the achievement of UHC [7,8].

As India struggles under the burden of diabetes and rising non-communicable diseases, the integration of cost-effective traditional systems, complementary and alternative medicine with the conventional medical systems, under Indian Ministry of AYUSH (Ayurveda, Yoga, Unani, Siddha, and Homeopathy) is inevitable, as it seeks to promote holistic health [9]. A cross-sectional study based on data collected from the National Health Interview Survey at Centre for Disease Control and Prevention in the United States has highlighted the prevalence of such approaches. In this study, yoga was recognized as one of the seven most frequently used complementary health approaches (CHA) among adults aged 65 or older, and it was concluded that 29.2% (11.7 million) of the older adult population used any of these seven CHAs [10]. This shows the increasing acceptance of complementary approaches among people. Telles et al., in a cross-sectional survey, had studied the characteristics of yoga practitioners in India in a manner similar to studies previously undertaken in the U.S.A. and Australia. Based on the response to the survey, it was found that yoga practitioners in India are predominantly males, with the chief reason to practice yoga being physical fitness [11] and not disease prevention. However, evidence suggests that yoga plays a pivotal role in changing the physiology of the body. Some of the yoga practices used in these studies included (a) abdominal stretching asanas (postures) that may help in the rejuvenation and regeneration of pancreatic cells beneficial to diabetes patients [12], (b) pranayama (breathing exercises), and (c) meditation that regulates the hypothalamic–pituitary–adrenal axis, resulting in decreased cortisol levels, heart rate, and heart rate variability [13,14]. 

When the world recognized 21 June [the summer solstice, the longest day of the year in the northern hemisphere] as the International Day of Yoga, the Ministry of AYUSH, India devised a Common Yoga Protocol for the promotion of positive health. This included loosening exercises followed by sitting, standing, and supine postures in combination with breathing exercises and meditation [15]. We wanted to know about health awareness in the Indian population, particularly with respect to perceptions of yoga (and not its subtypes) and the number of people practicing yoga in order to understand whether there is any knowledge–practice gap among Indians for a practice that originated in India. Besides, the implementation of yoga protocols, as public policy, requires evidence that there is not only positive perceptions about yoga but also an increased proportion of yoga practitioners in India. For example, in a school-based yoga program, which was developed and implemented in the U.S.A., about 5400 trained yoga instructors were employed to cover 940 schools to teach four basic aspects of yoga to school-going children [16]. Similar such policies and programs were developed in other states of the U.S.A and need to be developed in India as well. Consequently, the role of yoga, as mind–body training in schools and public health became critical in its implementation, as explained in the aforementioned report. 

Thus, it is becoming important to not only acquire data about the acceptability and perception about yoga in the population but also health care professionals in order to understand the knowledge–practice gap and availability of yoga resources in different zones of the country. This would help in establishing more yoga wellness centers and the orientation of health care workers to advocate its health-promoting effects. This would also provide the required evidence of the extent of acceptability of yoga with respect to its knowledge, acceptability, and practice before any new policy intervention. Thus, it is imperative that data from such a survey regarding yoga awareness, attitudes, and its practice are made available to lawmakers and scientists for research and translation. In view of this, the present study was designed to estimate the state of knowledge, attitude and practice of yoga (KAP-Y) for diabetes prevention and management. The results from our studies constitute the first-ever structured report of KAP yoga in Asia.

## 2. Materials and Methods 

This study was a nationally representative survey conducted across India. The respondents of this survey belonged to both rural and urban areas in all zones of India. This study was named in Hindi as *Niyantrita Madhumeha Bharata* (NMB) (or control diabetes in India) using a yoga-based lifestyle for the prevention and management of diabetes. 

This study was funded by the Ministry of Health and Family Welfare and the Ministry of AYUSH, Government of India, New-Delhi, coordinated by Indian Yoga Association (IYA) and assigned to Swami Vivekananda Yoga Anusandhana Samsthana (S-VYASA, Bengaluru), who is a member organization of IYA. The study was approved by the Institute Ethical Committee of Indian Yoga Association (vide Res/IEC-IYA/001 dt 16.12.16). Signed written informed consent was obtained from all participants. The approval date is 16 December 2016.

**Figure 1:**
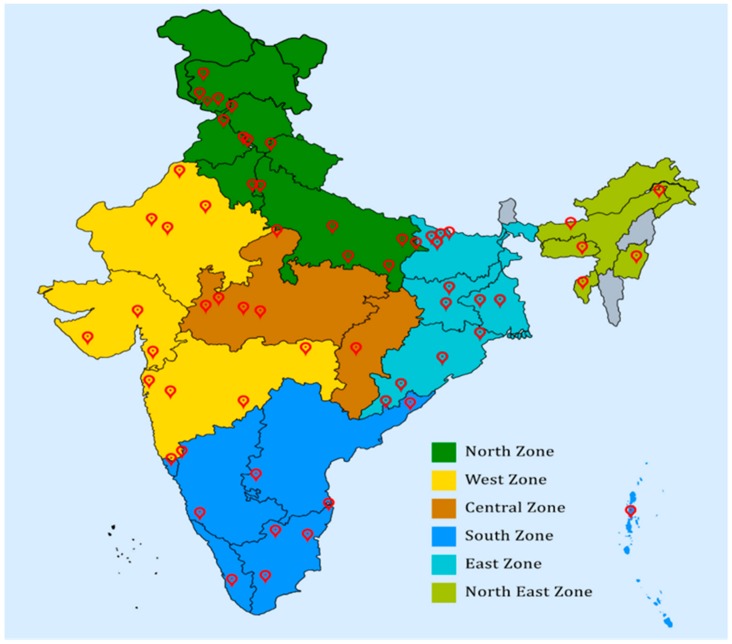
Representing six different zones of India.

Details of the methodology have been published as two articles [17,18]. In brief, in order to plan the survey, 65 districts from the 30 (out of 35 in India) most populous states and union territories from all six zones (Figure 1) were selected randomly depending on each state’s population density. Further, urban and rural clusters were identified from north, south, east, west, and central areas of each district using randomization. After identifying these clusters, the trained volunteers conducted the study in two phases of screening using a mobile app developed for the purpose by the international research advisory team. The first phase included a short questionnaire to identify individuals with high risk for diabetes and yoga awareness; the second phase included detailed data acquisition from those with high risk. Before the initiation of the study, all the volunteers undertook a six-day training explaining the content and manner of operationalization of the questionnaire, the importance of informed consents, and documentation of all the details in the questionnaire. The volunteers who had not undergone the training were not allowed to be a part of the study.

The first phase of the study included a door-to-door survey by knocking on all the doors across all the identified regions (urban and rural). The participants who consented were recruited for the study. At this time, they were not informed about the second phase of the study, neither were they compensated for their participation. This phase was a survey that consisted of the three-page item questionnaire, which was read out by interviewer in their native language or self-administered depending upon the convenience of the subject. This included demographic details of their socio-economic status (based on a modified Kuppuswamy scale] [19], Indian Diabetes Risk Score (IDRS) score [20], and yoga awareness. Three questions about KAP-Y were asked by reading the questionnaire. This became the basic conceptual framework for this manuscript. The yoga-related questions were as follows: (a) Do you think yoga can help in the prevention and management of Diabetes? (b) Do you think yoga can help change your lifestyle? (c) Do you practice yoga? (Practice of yoga implies the inclusion of standing, sitting, prone, and lying postures, deep breathing and/or meditation practice, if practiced previously in their lifetime or currently practicing.) As the subjects needed to be contacted again, the demographic details of the subjects were noted down; therefore, the option to retain the anonymity of participants was not given to subjects. Apart from the demographic details and the IDRS score, the rest of the questions are open-ended with ‘Yes’ or ‘No’ options. Therefore, it is unlikely that the administration of the questionnaire influenced the answers of the participants or introduced any perceivable bias.

### 2.1. Data Curation

**Figure 2:**
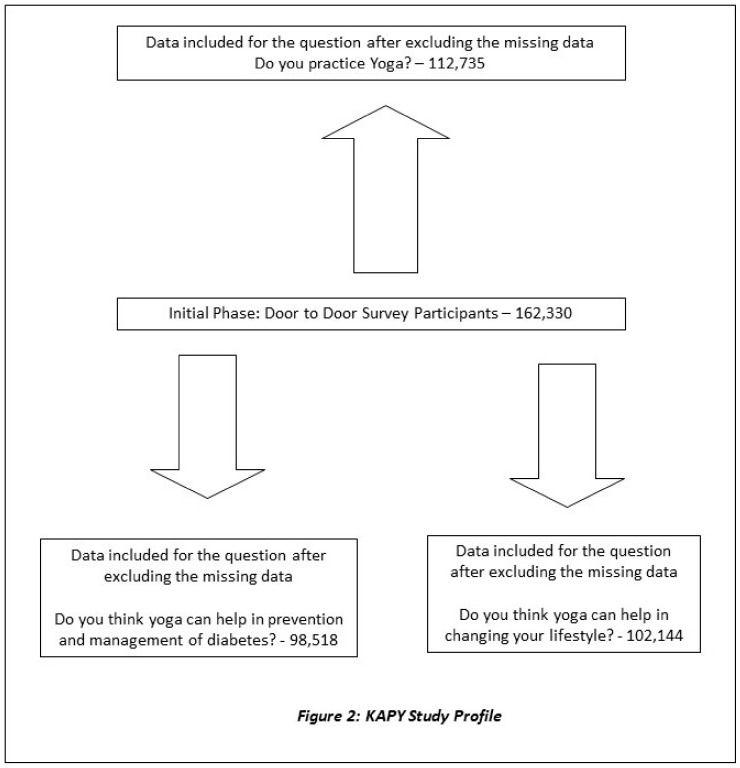
KAP-Y Study Profile. KAP-Y: state of knowledge, attitude and practice of yoga.

The data was simultaneously collected from all over the country, as outlined in Figure 1. Since it was a large data set, the uploaded data from the NMB Apps was cross-verified randomly with hard copies before analysis. The coding of the data was also centralized at S-VYASA. The curated data excluding the missing data are represented in Figure 2, which shows the study profile.

### 2.2. Statistical Analysis

To analyze the documented data, both descriptive statistics i.e., profile mapping and cross-tabulation were performed using the Statistical Package for Social Sciences (IBM Statistics for windows, SPSS v21.0) at S-VYASA, Bengaluru, India.

## 3. Results

A total of 162,330 participants were a part of the initial door-to-door survey and filled out the NMB questionnaire. As explained above, the questionnaire was administered by the volunteers recruited for the study.

### 3.1. Knowledge and Attitude about Role of Yoga in Changing Lifestyle

Table 1 shows the answers to question, “Do you think yoga can help with changing your lifestyle?” Most individuals (94,135/101,643) believed that yoga improves their lifestyle, out of which 49,586 were males and 44,549 were females. Upon classification as per various zones, central India (4442/102,144) revealed the popular belief that yoga improves lifestyle to a lesser extent as compared to the rest of India, and the highest is seen in south India (28,306/102,144). Furthermore, 43,420/99,272 participants of rural India and 48,413/99,272 participants of the urban India believed that yoga improves lifestyle. Both diabetic (9,859/10,367) and non-diabetic (80,952/87,785) participants also responded positively in this context (Table 1).

### 3.2. Perception of Yoga for Diabetes Prevention

Table 2 shows the answers to question, “Do you think yoga can help in the prevention and management of diabetes?” A vast majority of the participants (47,488 males and 42,614 females out of 98,518 participants) believed that yoga prevents and aids in the management of diabetes. A total of 9454/9930 participants with Type 2 Diabetes Mellitus (T2DM) and 77,635/85,414 participants with no diabetes reported that Yoga helps in preventing Diabetes (Table 2).

### 3.3. Yoga Practitioner Count and Characteristics Vary Across India

The study revealed that 11.8% (13,336/112,735) of people practice yoga and 88.2% (99,399/112,735) of people don’t practice yoga across India. Among the population practicing yoga, 7010/112.735 of males and 6233/112,735 of females are practicing yoga. Among various zones, minimal practice was noted from the east zone (971/112,735) and the highest practice was noted from the north zone (4567/112,735). It is noted that 6939/109,888 of the urban population and 6277/109,888 of the rural population was found to be practicing yoga. The difference in the number of yoga practitioners among the diabetic population (1789/11,342) versus non-diabetic population (11,042/98,776) was noted (Table 3).

## 4. Discussion

This pan India cluster sample survey constituted 162,330 participants, revealing 11.8% of the population to be practicing yoga. There was an almost equal prevalence of yoga practice among various categories—the males and females, urban, new diabetics, pre-diabetics, and age group (60–79 years) besides the upper–middle class socio-economic status.

This study provides a significant insight regarding the widely held views of yoga’s efficacy. This is evident from the results of knowledge and attitude sampling. Although it shows that it is perceived as useful for lifestyle modifications (92.6%), the proportion of those adopting the practice of yoga is not comparable (11.8%). This knowledge–practice gap can be due to various reasons. The comparison of our results with data across the world shows that there are a comparable proportion of yoga practitioners. However, other surveys were not sampled as door to door in a cluster design and were not carried out for such a large sample. The other methods of surveys included emails, online, and convenient sampling, all of which lack the reliability, interface, and rigor that the door-to-door survey provides.

The increased awareness could be partly because of yoga’s origins and popularity in India and partly because the current Indian PM’s role modeling of yoga for health on each International Day of Yoga may have aligned this cultural practice with health and wellness.

### 4.1. Germany

A representative sample size of 2041 participants from Germany in the age group of more than 14 years were interviewed, and it was found that 68 participants (3.3%) acknowledged practicing yoga when quizzed at the time of interview, while 241 participants (11.8%) practiced yoga prior to the interview. It was estimated that around 15.7 million Germans either practice yoga or are interested in practicing yoga. They concluded that the lifetime prevalence of yoga practice in Germany was 15.1% with a point prevalence of 3.3%. A greater popularity of yoga was found linked with females, better education, and employment status. The authors reported that their survey had the limitation of retrospective analysis and an inability to account for the different components and quality of yoga. When compared with our study, the sample size is much smaller, and the prevalence of practicing yoga is also not comparable [21]. The quality of yoga being practiced in the absence of popular yoga gurus or institutions in this country is also not clear.

### 4.2. United Kingdom (UK)

Annual cohort studies conducted during the period of 1997 to 2008 in the United Kingdom (UK) included physical activity questions. The data were collected during household visits via the interviews at three time points (1997 to 1999; 2003 to 2004; and 2006/2008) and were asked with an open-ended question about the practice of yoga in the last four weeks. A total of 81,090 participants (1997–1999: 38,409; 2003–2004: 27,580, and 2006/2008: 15,101) responded to the interviews. The proportion of females, educated individuals, and the working class was higher with a point prevalence to be at 0.46% (175 respondents during 1997–1999), 0.94% (260 respondents during 2003–2004), and 1.11% (168 participants during 2006/2008), which shows an increasing trend [22]. However, this was a long duration study in which the knowledge, attitude, and practice may have changed over time. Moreover, the sample size was far smaller than that of the current study from India.

### 4.3. Australia

In a national cross-sectional survey of the Australian women, where data was collected from the Australian Longitudinal Study on Women’s Health (ALSWH), a self-reporting questionnaire was sent to randomly selected women. The response rate was 42% to 56%, which included a total of 28,695 women on three different cohorts based on birth year, 9151 women in 1946–1951; 8200 women in 1973–1978; 11,345 women in 1989–1995. It was found that the prevalence of practicing yoga/meditation is about 20.7% (1946–1951 cohort), 21.7% (1973–1978 cohort) and 29% (1989–1995 cohort), respectively. It is worth mentioning from the cohorts that the popularity of yoga is prominent among the younger population, but the regularity in the practice is more pronounced in the older population with a prevalence of 11.1% (1946–1951 cohort), 7.7% (1973–1978 cohort), and 9.2% in (1989–1995 cohort). The major limitation of this study was that a single open-ended question was asked on both the practice of either yoga or meditation; therefore, the results stated in the study cannot inform us of whether the prevalence is only for yoga, for meditation, or for both [23]. 

### 4.4. USA

This pattern was also found to be consistent with National Health Interview Survey (NHIS-2012), which revealed that 31 million adults had practiced yoga ever, whereas 21 million adults practiced yoga in the previous 12-month period with a lifetime prevalence of 13.2% and 12-month prevalence being 8.9%. The popularity was more among females, educated individuals, and the working class [24]. This survey was not as large as the current one in terms of duration of operationalization and method of execution (door to door). Interestingly, there was a increase in the awareness of yoga in U.S.A from the year 1998, where it was assessed that 15 million adults practiced yoga, whereas 7.4 million adults practiced yoga in the previous 12-month period with a lifetime prevalence of 7.5% and 12-month prevalence being 3.8% [25] to 13.2% lifetime prevalence and 8.9% of 12-month prevalence [24]. It is also interesting to note that prevalence studies for the practice of meditation in the U.S.A adult population indicated greater prevalence among females and those who were college educated. A total of 9.3 million adults practiced meditation in the previous 12-month period with a lifetime prevalence of 5.2% and 12-month prevalence being 4.1% [26]. The overall practice of yoga has increased in the U.S.A.; however, the use of medicalized/referral-based yoga has decreased, as per a study by Patwardhan and Lloyd [27].

### 4.5. India

As cited before in this paper, the survey conducted by Telles et al. [11] studied the educational background of 5157 yoga practitioners in India and reported them to be high school graduates with more male practitioners (67.3) as compared to females (32.7). This was the first study in an Asian population that has shown a higher prevalence in males than females when compared to studies in the west. This is similar to our study in which males were marginally higher than females.

From the current study, we note that the prevalence of the yoga in India is 11.8%, and 91.5% of participants believe that yoga yields health benefits such as the control of T2DM. This is higher compared to Western countries. This may be due to the cultural origins of yoga, which is aligned to Indian traditions and lifestyle, manifesting as yoga literature in Sanskrit and Hindi. Apart from these, there are many yoga role models such as Baba Ramdev promoting yoga from many years through various platforms such as television and social media. Even the Prime Minister of India, Narendra Modi advocates yoga [28] and its benefits. This has also promoted yoga in the Indian community. After the United Nations (UN) adopted 21 June as the International day of Yoga, it has since been adopted by other Western countries [21,22,24].

### 4.6. Knowledge of Yoga for Health Benefits

In our present study, we noticed that 92.6% of the participants, irrespective of their gender, believed that yoga helps in superior health outcomes. The study may provoke further investigation about what roles poorer self-regulation and implicit attitudes play on the reduced practice of yoga in contrast to nationwide popular perception that it prevents diabetes. The practice of yoga also requires training by expert yoga practitioners. The number of expert practitioners is believed to be limited to only 2034, in which 1360 practitioners are limited to the Tamil Nadu and Karnataka states of India [29]. This shows the need for a greater number of yoga practitioners and training schools in the country.

In contrast, allopathic practice, which is widely prevalent, usually shows a general compliance of about 30–50% [30]. Even though India is open to complementary and alternative medicine (CAM) practice, it was noted in a study done by Roy et al. (2015) that only 28% of the patient population practice CAM [31].

### 4.7. Yoga as CAM

A cross-cultural comparative analysis was carried out to examine the health-seeking behavior of university students from New Delhi (India), Newcastle upon Tyne (United Kingdom) and Atlanta (United States). The Indian students opted for CAM over allopathic medicine, and nutrition-based approaches [supplements, etc.] were found to be most prevalent among the students. Considering the unique health care systems of the three countries, it was concluded that 55% of Indian students considered CAM as a more affordable approach than routine healthcare practices [32].

According to a KAP study done by Kong et al. (2013) among the medical practitioners, it was observed that 71% of medical practitioners feel that CAMs are effective with allopathic treatment [33]. According to Roy et al. (2015) even though 58% of the doctors use CAMs for themselves, only 37% recommend their patients and enquire about the utilization of them. This highlights the importance of creating awareness among the medical practitioners [31].

## 5. Limitations

Since the data obtained was through recall and self-reporting, there may have been bias in recalling the individual practice of yoga. The study has shown missing data due to some of the following reasons. (a) The completion of the entire questionnaire was not mandatory, as the subjects in the survey could choose not to answer some of the questions. (b) In some of the rural and tribal areas of India, the language was a significant barrier, and the missing data was reported. The study would have been benefitted by asking the question, “Would you like to practice yoga?” This question would have explained the attitude of yoga among the India population more convincingly. However, belief that yoga in lifestyle modification and the prevention and management of diabetes mellitus in all the age groups provides an indirect evidence of the positive attitude of yoga in India. Despite these limitations, this is the largest study on KAP-Y use in Asia.

## 6. Conclusions

The study highlights the variation in prevalence of yoga practice based on the demographic characteristics and concludes that 11.8% of the Indian population practices yoga across both rural and urban areas. However, further studies are required to identify the therapeutic or preventive benefits of yoga in various clinical conditions along with an evidence-based inclusion of yoga in clinics, which may partially bridge the chasm that exists between the knowledge and practice of yoga. The study also recommends education and training for yoga theory and practice for practitioners as well as physicians. In addition, research studies will help yoga be integrated for public education, training, and the management of diseases.

## Figures and Tables

**Table 1:** KAPY 2017.

Do you Think Yoga Can Help with Changing Your Lifestyle?								
Characteristics		YES		NO		Total		*p*-Value
		N	%	N	%	N	%	
**Gender**	Male	49,586	92.8	3856	7.2	53,442	100	<0.0001
	Female	44,549	92.4	3652	7.6	48,201	100	<0.0001
	**Total**	**94,135**	**92.6**	**7508**	**7.4**	**101,643**	**100**	
**Zone**	East	13,247	87.3	1935	12.7	15,182	100	<0.0001
	West	17,680	95.4	851	4.6	18,531	100	<0.0001
	North	25,046	96.0	1037	4.0	26,083	100	<0.0001
	South	28,306	98.7	378	1.3	28,684	100	<0.0001
	Central	4442	61.3	2808	38.7	7,250	100	<0.0001
	Northeast	5859	91.3	555	8.7	6,414	100	<0.0001
	**Total**	**94,580**	**92.6**	**7564**	**7.4**	**102,144**	**100**	
**Area**	Rural	43,420	92.1	3721	7.9	47,141	100	<0.0001
	Urban	48,413	92.9	3718	7.1	52,131	100	<0.0001
	**Total**	**91,833**	**92.5**	**7439**	**7.5**	**99,272**	**100**	
**Self-Reported Status of T2DM**	No T2DM	80,952	92.2	6833	7.8	87,785	100	<0.0001
	Known T2DM	9859	95.1	508	4.9	10,367	100	<0.0001
	**Total**	**90,811**	**92.5**	**7341**	**7.5**	**98,152**	**100**	
**Age**	20-39	45,028	92.1	3836	7.9	48,864	100	<0.0001
	40-59	35,589	92.7	2822	7.3	38,411	100	<0.0001
	60-79	12,667	93.6	863	6.4	13,530	100	<0.0001
	Above 80	257	95.9	11	4.1	268	100	<0.0001
	**Total**	**93,541**	**92.5**	**7532**	**7.5**	**101,073**	**100**	
**Socio-economic level**	Low	160	97.0	5	3.0	165	100	<0.0001
	Upper low	7626	93.3	545	6.7	8,171	100	<0.0001
	Lower–middle	30,318	91.8	2717	8.2	33,035	100	<0.0001
	Upper–middle	17,003	92.4	1390	7.6	18,393	100	<0.0001
	Upper	885	87.8	123	12.2	1,008	100	<0.0001
	**Total**	**55,992**	**92.1**	**4780**	**7.9**	**60,772**	**100**	

**Table 2:** KAPY 2017.

Answers To: “Do You Think Yoga Can Help in the Prevention and Management of Diabetes?”								
Characteristics		YES		NO		Total		*p*-Value
		N	%	N	%	N	%	
**Gender**	Male	47,488	91.5	4428	8.5	51,916	100	<0.0001
	Female	42,614	91.4	3968	8.6	46,602	100	<0.0001
	**Total**	**90,102**	**91.5**	**8416**	**8.5**	**98,518**	**100**	
**Self-Reported Status of T2DM**	No T2DM	77,635	90.9	777	9.1	85,414	100	<0.0001
	Known T2DM	9454	95.2	476	4.8	9930	100	<0.0001
	**Total**	**87,089**	**91.3**	**8255**	**8.7**	**95,344**	**100**	

**Table 3:** Socio-demographic distribution of practitioners of yoga in India.

Characteristics		Practicing Yoga		Not Practicing Yoga		Total		*p*-Value
		N	%	N	%	N	%	
**Overall**		13,336	11.8	99,399	88.2	112,735	100	
**Gender**	Male	7010	11.9	52,035	88.1	59,045	100	<0.0001
	Female	6233	11.7	46,969	88.3	53,202	100	<0.0001
**Zone**	East	971	5.3	17,219	94.7	18,190	100	<0.0001
	West	2057	10.5	17,551	89.5	19,608	100	<0.0001
	North	4567	17.2	21,901	82.7	26,468	100	<0.0001
	South	3732	14.5	22,093	85.5	25,825	100	<0.0001
	Central	1096	10.3	9575	89.7	10,671	100	<0.0001
	North east	913	7.6	11,060	92.4	11,973	100	<0.0001
	**Total**	**13,336**	**11.8**	**99,399**	**88.2**	**112,735**	**100**	
**Area**	Rural	6277	11.9	46,414	88.1	52,691	100	<0.0001
	Urban	6939	12.1	50,258	87.9	57,197	100	<0.0001
	**Total**	**13,216**	**12.0**	**96,672**	**88.0**	**109,888**	**100**	
**Self-Reported Status of T2DM**	No T2DM	11,042	11.2	87,734	88.8	98,776	100	<0.0001
	Known T2DM	1789	15.8	9553	84.2	11,342	100	<0.0001
	**Total**	**12,831**	**11.7**	**97,287**	**88.3**	**110,118**	**100**	
**Age**	20–39	6256	11.8	46,934	88.2	53,190	100	<0.0001
	40–59	5105	11.8	37,987	88.2	43,092	100	<0.0001
	60–79	1839	12.1	13,296	87.9	15,135	100	<0.0001
	Above 80	20	6.6	282	93.4	302	100	<0.0001
	**Total**	**13,220**	**11.8**	**98,499**	**88.2**	**111,719**	**100**	
**Socio-economic level**	Low	17	10.3	148	89.7	165	100	<0.0001
	Upper low	1004	11.7	7575	88.3	8579	100	<0.0001
	Lower–middle	4577	12.6	31,655	87.4	36,232	100	<0.0001
	Upper–middle	2853	13.5	18,207	86.5	21,060	100	<0.0001
	Upper	121	10.5	1035	89.5	1156	100	<0.0001
	**Total**	**8572**	**12.8**	**58,620**	**87.2**	**67,192**	**100**	
